# On the Bleaching of Yellow Bees-Wax

**Published:** 1844-12-14

**Authors:** M. Ingenohl


### ON THE BLEACHING OF YELLOW BEES-WAX.

BY M. INGENOHL.

A very expeditious method of bleaching wax is by the application of nitric acid in the following manner: A pound of yellow bees-wax is melted, and freed by straining from impurities; 2 oz. of nitrate of soda are then added to it, and subsequently by degrees 1 oz. of sulphuric acid, which has been previously diluted with at least nine parts of distilled water; the whole mass is kept warm, and constantly stirred with a glass rod. The vessel must be spacious, especially very high, as the mass rises considerably. When the whole of the dilute sulphuric acid has been added, it is allowed to cool somewhat, the vessel filled with boiling water, well agitated and set aside. The wax-cake is removed, and conveyed into boiling water until this no longer removes any sulphate of soda from it, and consequently does not produce any turbidness in a solution of chloride of barium; the wax is then perfectly white. By the melting in water, every trace of nitric acid is removed, which, were it present, would be decomposed on exposure to light into oxygen and nitrous acid, and would thus impart a reddish colour to the wax.—*Chemical Gazette.*

